# High-Temperature Atomic Layer Deposition of GaN on 1D Nanostructures

**DOI:** 10.3390/nano10122434

**Published:** 2020-12-05

**Authors:** Aaron J. Austin, Elena Echeverria, Phadindra Wagle, Punya Mainali, Derek Meyers, Ashish Kumar Gupta, Ritesh Sachan, S. Prassana, David N. McIlroy

**Affiliations:** 1 Department of Physics, Oklahoma State University, Stillwater, OK 74078-3072, USA; aaron.j.austin@okstate.edu (A.J.A.); elena.echeverria@okstate.edu (E.E.); pwagle@okstate.edu (P.W.); pmainal@okstate.edu (P.M.); Derek.meyers@okstate.edu (D.M.); spa.phy@psgtech.ac.in (S.P.); 2 School of Mechanical and Aerospace Engineering, Oklahoma State University, Stillwater, OK 74074-3072, USA; ashish.gupta10@okstate.edu (A.K.G.); rsachan@okstate.edu (R.S.); 3 Center for Surface Science, Department of Physics, PSG College of Technology, Coimbatore 641004, India

**Keywords:** gallium nitride, atomic layer deposition, nanosprings, functional coatings, custom ALD reactor

## Abstract

Silica nanosprings (NS) were coated with gallium nitride (GaN) by high-temperature atomic layer deposition. The deposition temperature was 800 °C using trimethylgallium (TMG) as the Ga source and ammonia (NH_3_) as the reactive nitrogen source. The growth of GaN on silica nanosprings was compared with deposition of GaN thin films to elucidate the growth properties. The effects of buffer layers of aluminum nitride (AlN) and aluminum oxide (Al_2_O_3_) on the stoichiometry, chemical bonding, and morphology of GaN thin films were determined with X-ray photoelectron spectroscopy (XPS), high-resolution x-ray diffraction (HRXRD), and atomic force microscopy (AFM). Scanning and transmission electron microscopy of coated silica nanosprings were compared with corresponding data for the GaN thin films. As grown, GaN on NS is conformal and amorphous. Upon introducing buffer layers of Al_2_O_3_ or AlN or combinations thereof, GaN is nanocrystalline with an average crystallite size of 11.5 ± 0.5 nm. The electrical properties of the GaN coated NS depends on whether or not a buffer layer is present and the choice of the buffer layer. In addition, the IV curves of GaN coated NS and the thin films (TF) with corresponding buffer layers, or lack thereof, show similar characteristic features, which supports the conclusion that atomic layer deposition (ALD) of GaN thin films with and without buffer layers translates to 1D nanostructures.

## 1. Introduction

Atomic layer deposition (ALD) is an extremely valuable technique for growing conformal and precision ultrathin films for transistors, light emitting diodes [1,2], capacitors [3,4], solar cells [5,6], as well as different types of storage media, such as dynamic random-access memory (DRAM) and hard disk drives (HDD) [7,8], and recently solid-state batteries [9,10]. Due to the excellent surface conformation of ALD coatings, it is ideally suited for coating complex nanostructures. Furthermore, ALD for functional nanostructure coatings can improve the performance of devices or add stability to complicated structures [11,12,13,14,15,16]. The desire to develop nanostructures with technologically important coatings like GaN dictates that ALD processes be developed specifically for coating complex nanostructures. The same can be said for ALD deposition systems. Many commercially available ALD systems are not designed to operate at high temperatures. This is necessary for obtaining high-quality GaN and other more exotic materials. With this in mind, we begin by exploring existing reviews of ALD to elucidate the advantages, disadvantages, and deposition system designs [17,18].

Conventional thermal ALD systems operate at temperatures less than 350 °C (see Ref. [19] and references therein), mainly due to the desire to grow at low temperatures and the use of O-ring chamber seals. Studies of ALD growth of ZnO, GaN, and Al_2_O_3_ show that surface, electrical, and optical characteristics are dependent on the growth temperature [20,21,22]. Having a larger window of growth temperatures can significantly improve materials properties, which is important to industries that utilize ALD or would like to. Furthermore, higher temperatures can enable the use of precursors that require higher temperatures to react with a reducer. The alternative to higher temperature decomposition of precursors is to incorporate plasma assisted decomposition, typically referred to as plasma enhanced ALD (PEALD) [18,23]. The diversity of precursors that can be used with PEALD arises from the fact that the plasma pre-dissociates the precursor, thereby increasing its reactivity with the surface. Having enhanced surface reactions leads to further reductions in substrate temperatures. This is important when growing on substrates that cannot tolerate high temperatures, such as plastics/polymers [24,25,26,27]. Unfortunately, PEALD adds complexity to the system with the addition of a plasma generator, additional gases and flow controllers. Lastly, one has to consider the accelerated deposition rates associated with PEALD and its effect on film stoichiometry and morphology.

The goal of the present work is coating 1D nanostructures with high quality GaN by ALD, but this requires higher deposition temperatures than conventional ALD systems [22,28,29,30,31]. To this end, a high-temperature ALD (HTALD) deposition system capable of temperatures greater than 800 °C has been developed and is, therefore, a part of the work presented herein. The foundation of the design is the construction of a reactor inside a vacuum chamber, as opposed to the reactor being the vacuum chamber. This approach eliminates the use of O-ring seals that limit the deposition temperature. Note that the system is designed for small samples, as opposed to large samples like whole Si wafers. Consequently, it is not optimized for film thickness uniformity across a large substrate. This is not to say that it could not be adapted for large substrates if the need arose. Lastly, the design incorporates a halogen bulb as the heat source. Halogen bulbs with a power of 1000 W are readily available and only cost a few US dollars and have proven to be highly durable in the present system.

Gallium nitride is a desirable material for coating 1D nanostructures because of its ability to operate at high temperatures [32] and its superior power density relative to Si [33]. The majority of studies of ALD deposition of GaN are at low temperature (<500 °C) and/or plasma enhanced ALD, with the exception of the work by Kim et al. [34] using gallium chloride (GaCl_3_) and ammonia (NH_3_) as the sources. In the vain of this special issue, NS were chosen as the test platform because their morphology is ideal for exploring a processes ability to produce conformal coatings, the limits of its ability to control the thickness of the coating, and the effects of surface morphology on the crystallinity of the coating in the presence of high levels of stress and strain. Silica nanosprings are a versatile 1D nanostructure that with the appropriate coating, such as with ZnO, have been utilized in chemical sensors [35], as a catalyst support structure for biofuel synthesis [36], and as a biomimetic material [37], to name a few. This study also examines the effects of buffer layers on its morphology and electrical properties of the GaN coating. Past reports on the growth of GaN thin films have demonstrated that the quality of the films improves with the addition of AlN buffer layers in the form of superior physical and electrical properties [38,39,40]. Specifically, the studies have shown that buffer layers relieve strain in thin films and improves their electrical properties [40,41,42,43,44], which will be put to the test with silica nanosprings as the substrate. A detailed description of the custom HTALD system is presented, followed by the characterization of GaN grown at 800 °C on NS and Si substrates with and without AlN and Al_2_O_3_/AlN buffer layers.

## 2. High-Temperature Atomic Layer Deposition System Design

**Figure 1:**
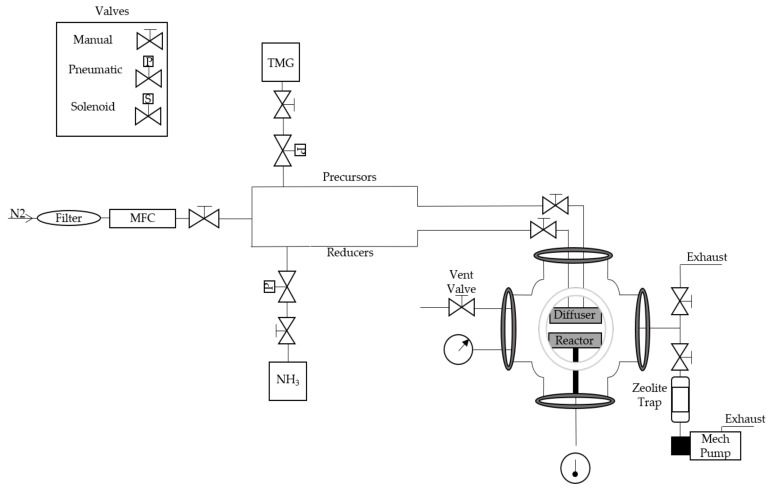
A schematic of the high-temperature atomic layer deposition (ALD) gas handling system, the vacuum chamber and vacuum pumping system.

The HTALD system design is a reactor within a vacuum system, as opposed to an integrated reactor/vacuum system (Figure 1). With appropriate heat shielding, this allows the reactor to reach temperatures in excess of 800 °C, while keeping the walls of the vacuum chamber well below 200 °C. This facilitates the use of ISO flanges which have viton O-rings that otherwise will melt at temperatures greater than 200 °C. Furthermore, ISO flanges enable quick access to the ALD reactor and quick turnaround when loading or unloading samples. The reactor is a stainless steel six-way spherical cross with ISO 250 flanges (Figure 1). The spherical cross design reduces the proximity of the chamber walls to the reactor. Stainless heat shielding is placed around the reactor to further protect the O-rings, with the added benefit of reflecting heat back to the reactor. A schematic of the gas delivery system is shown in Figure 1. Nitrogen (N_2_) carrier gas passes through an in-line oxygen filter (Oxiclear RGP-R1-500) and a 0–100 sccm mass flow controller (MFC). Pneumatic valves are sequenced to deliver reactants at precise rates. These valves can be in line with each bottle or gas bottles of similar families. The system has two separate gas lines, one for precursors and one for reducers. All of the source bottles and lines are stainless steel (SS) which are placed in an exhaust cabinet, where each source bottle is sealed with an in line manual valve. The lines are routed to the top of the chamber and gas is delivered from above, similar to a showerhead. 

**Figure 2:**
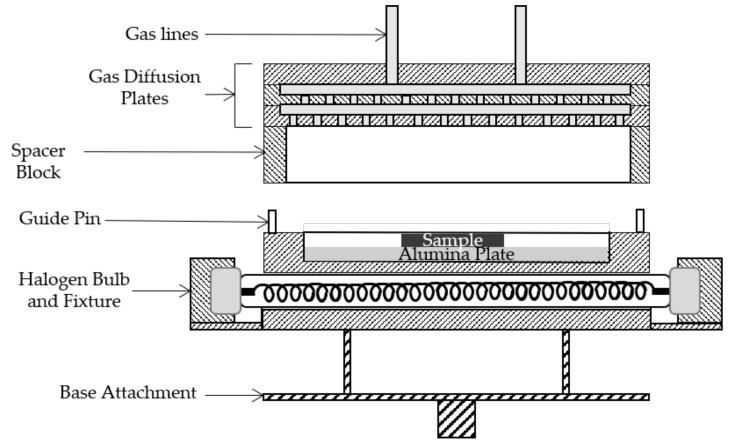
Schematic of the a high-temperature ALD (HTALD) Reactor. The top element is the movable gas diffuser assembly where the bottom element is the stationary heater assembly.

A schematic of the reactor is presented in Figure 2. It consists of a stationary bottom component (sample heater assembly) that houses the heater and supports the sample and a movable top component (gas diffuser assembly) with two gas inlets at the top. The sample heater assembly is stainless steel (SS) and utilizes a 1000 W halogen bulb with a porcelain fixture that supports the bulb and keeps it from coming into contact with the walls of the assembly. A type K thermocouple is attached to the inner wall of the reactor near the sample. The sample is supported by an alumina plate placed a few millimeters above the halogen bulb. The alumina plate enhances absorption of IR emission from the bulb and has the benefit of minimal outgassing up to 1500 °C. The gas diffuser assembly consists of two diffuser plates with offset hole patterns to ensure uniform dispersion of the gases and a 3 cm spacer. The spacer provides space for the gases to expand upon exiting the diffuser for better uniformity of the film, as well as keeps the diffuser from overheating, which can result in breakdown of the TMG precursor inside the diffuser. The gas diffuser assembly is attached to a linear translator that allows it to be lowered into firm contact with the sample heater assembly to form a tight but non-vacuum seal. This allows the gases to escape the reactor into the surrounding vacuum chamber and are subsequently pumped away. Alignment pins on the heater assembly ensure proper alignment and seating of the gas diffuser assembly to the heater assembly. 

## 3. Growth Details and Characterization

Nanosprings grow via a modified vapor-liquid-solid growth mechanism, as reported by McIlroy et al. [45,46,47,48,49], where we refer readers to the references for the details. p-type Si(100) substrates were used for all the samples in this study, with or without silica NS mats. The Si(100) substrates were cleaned using the RCA Standard Clean process (15 min) developed by Kern in 1965 [50]. Trimethylgallium (TMG) was the Ga source and NH_3_ (99.99% Airgas) was used as the reactive nitrogen source. The TMG source was maintained at room temperature during deposition. A constant flow of 20 sccm of N_2_ (99.998% Airgas) through an oxygen filter was maintained to the reactor throughout deposition and the chamber pumping was throttled down to achieve a N_2_ chamber pressure of 500 mTorr. Gallium nitride deposition temperature for all of the samples was 800 °C. A single ALD cycle consisted of a 25 ms TMG dose: 10 s N_2_ purge: 3 s NH_3_ dose: 90 s N_2_ purge. ALD of AlN and Al_2_O_3_ were deposited in both a thermal and plasma assisted ALD system from Okyay Technologies. Aluminum oxide was deposited at 100 °C using trimethylaluminum (TMA) and Hyrdogen Peroxide (H_2_O_2_). Aluminum nitride was deposited at 170 °C using TMA and a N_2_ plasma as the reactive nitrogen source. For Al_2_O_3_, a cycle consisted of a 60 ms TMA dose: 10 s N_2_ purge: 1 s H_2_O_2_ dose: 10 s N_2_ purge: pumped down to the base pressure (~125 mTorr). AlN cycles consisted of a 50 ms TMA dose: 10 s N_2_ purge: 10 s N_2_ plasma dose: pumped down to base pressure (~1.5 Torr).

The film thickness of the samples was determined with variable angle spectroscopic ellipsometry (J.A. Woollam VASE Ellipsometer). X-ray photoelectron spectroscopy (XPS) was performed on the TF samples in an ultra-high-vacuum (UHV) system with a base pressure of 6.0 × 10^−10^ Torr. XPS spectra were acquired for as-grown and sputtered thin films. Samples were sputtered with Ar^+^ at 5 × 10^−5^ Torr for 15 min. The XPS spectra were acquired using the Mg-Kα emission line from a dual anode X-ray source (Physical Electronics XR 04-548) operated at 300 W and an incident angle of 54.7°. The kinetic energy of the photoelectrons were analyzed with an Omicron EA 125 hemispherical electron energy analyzer with a resolution of 0.02 eV. High-Resolution X-ray diffraction (HRXRD) spectra was acquired with a Cu-Kα radiation source. Scanning Electron Microscopy (SEM) micrographs were captured using an EI Quanta 600 field emission gun ESEM with Bruker EDS and HKL EBSD. Transmission Electron Microscopy (TEM) and Selected Area Electron Diffraction (SAED) of the coated NS was performed in a JEOL JEM-2100 transmission electron microscope with an EVEX X-ray microanalysis system. The spot size resolution for SEM was between 2–3 spot resolution and the voltage between 10–15 kV. Atomic Force Microscopy (AFM) imaging of the surfaces of GaN TF was obtained with a Nanosurf Easyscan 2 benchtop AFM. All of the electrical measurements were acquired on GaN TF and GaN coated NS substrates. I-V curves were acquired using a Keithley 2400 source/meter under dark and 470 nm illuminated conditions, where indium beads were used as contacts. DC resistivity measurements at room temperature and under dark conditions were acquired using a four-point probe setup, where the resistivity was calculated using Equation (1):

(1) $$\rho \;=\;\left(\frac{\pi }{2}\right)*\left(\frac{V}{I}\right)*d*k$$

where *d* is the film thickness and *k* is a correction factor (=ln(2)) that is based on the relationship between the diameter of the tips of the probes and diameter of the wafer. 

## 4. Results and Discussion

**Figure 3:**
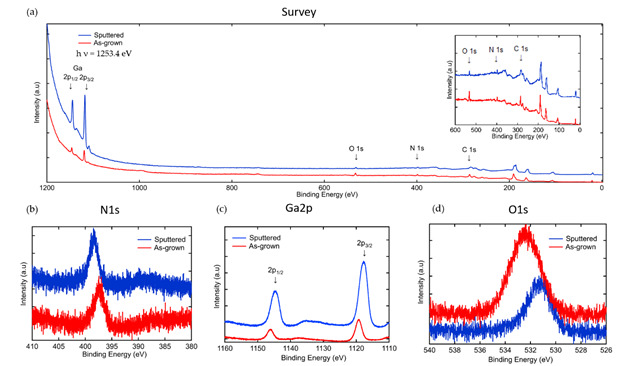
X-ray photoelectron spectroscopy (XPS) spectra comparing as-grown and sputtered GaN thin film grown at 800 °C, including (**a**) a survey scan with inset from 0 to 600 eV and core level states of (**b**) N 1s, (**c**) Ga 2p, and (**d**) O 1s.

X-ray photoelectron spectroscopy was performed to determine the elemental stoichiometry of the GaN. Figure 3a is a survey scan of the as-grown and sputtered GaN/Si(100), where peaks corresponding to Ga, C, and O are observed. The absence of the C 1s core level state in the survey scan (inset in Figure 3a) of the sputtered surface demonstrates that C was only at the surface or that the subsurface C concentration is below the resolving power of XPS. The presence of C is attributed to the methyl groups of TMG. High-resolution normalized scans of the N 1s, Ga 2p, O 1s, for the as-grown and sputtered film are displayed in panels (b–d) in Figure 3. With sputtering we see that the intensity of the Ga 2p core level states increase, the intensity of the N 1s remains constant, and the O 1s decreases. The reduction of the intensity of the O 1s core level state, but not its elimination like the C 1s, suggests that O is present in the GaN. The higher binding energy of the O 1s core level state of the as-grown sample in Figure 3b, is attributed to Ga-O bonding on the surface, while the O 1s binding energy of 532.2 eV after sputtering is attributed to chemisorbed oxygen or hydroxyl species [51,52,53,54]. The oxygen in the bulk is considered to be an unintentional n-type doping [55] and its impact on the electrical properties of the GaN TF and GaN coated NS will be discussed. The binding energy of the Ga 2p_1/2_ and Ga 2p_3/2_ core level states at 1146.2 eV and 1119.2 eV, respectively, shift to 1144.8 eV and 1117.8 eV with sputtering. The binding energies of the Ga 2p core level states for the sputtered sample are in agreement with values reported in the literature [34,56,57]. Figure 3d is the N 1s core level, where a shift of the binding energy from 397.1 to 398.5 eV is observed after sputtering. This shift of the N 1s core level state to higher binding energy is attributed to N dangling bonds [58,59].

**Figure 4:**
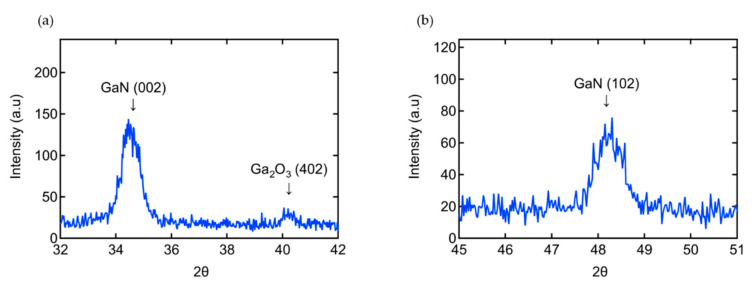
High-resolution x-ray diffraction (XRD) of 13 nm GaN thin films on Si (100) of (**a**) the GaN (002) peak and a small β-Ga_2_O_3_ peak and (**b**) the GaN (102) peak.

High-resolution XRD of the GaN TF is shown in Figure 4. Diffraction peaks are observed at 34.58° and 48.25° and correspond to the (002) and (102) planes of GaN, respectively [34,60,61,62]. A small peak at 40.20° corresponds to β-Ga_2_O_3_ (402) and is consistent with the XPS analysis [63]. Based on the absence of other GaN peaks and the relative intensity of the (002) to the (102), we have concluded the preferred growth is in the [0001] direction. The absence of other GaN planes precludes the formation of a large fraction of crystallites. The presence of the (102) plane has been attributed to high-temperature deposition that results in the formation of a small fraction of well-developed crystallites [64]. An alternative explanation is stress/strain induced dislocations [57,65].

**Figure 5:**
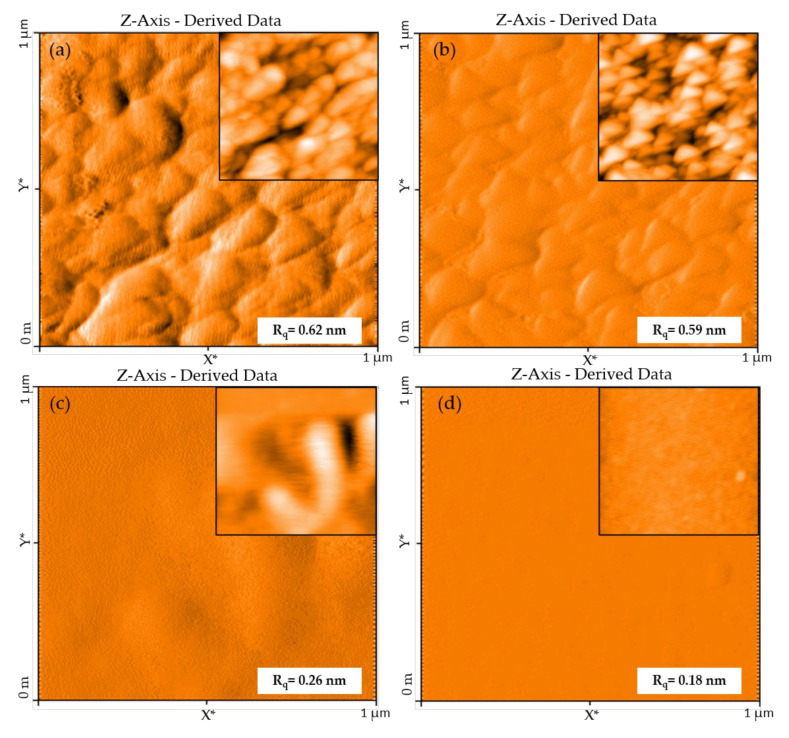
Atomic force microscopy (AFM) of GaN/Si(100) thin films grown at 800 °C with (**a**) 60 nm GaN (**b**) 13 nm GaN (**c**) 13 nm GaN with AlN buffer and (**d**) 13 nm GaN with Al_2_O_3_/AlN buffer. Line fits are included in the upper right-hand corner of each image.

Atomic force microscopy has been used to determine the RMS roughness of the surface of the GaN thin films. Displayed in Figure 5 are AFM images of GaN of various thicknesses and buffer layers. The root-mean-square roughness (R_q_) of GaN(60 nm), GaN(13 nm), GaN(13 nm)/AlN and GaN(13 nm)/AlN/Al_2_O_3_ are 0.62 nm, 0.59 nm, 0.26 nm, and 0.18 nm, respectively. The surface topography of the GaN films without buffer layers (Figure 5a,b) have equivalent RMS surface roughness, independent of their thicknesses. Furthermore AFM shows that that they have similar surface morphology and that the films are continuous and consistent with a study by Kim et al. of ALD deposition of GaN [34]. The inclusion of buffer layers of AlN and Al_2_O_3_/AlN reduces the RMS roughness to 0.26 nm and 0.18 nm (Figure 5c,d), respectively, due to a reduction in grain formation. This, in turn, is due to the smaller lattice mismatch of Al_2_O_3_ and AlN with GaN, as compared on Si(100) [44,66]. The lattice mismatch between GaN and Si is further exacerbated by their different coefficients of thermal expansion, which leads to higher internal stress [67]. The impact of mitigating stress with buffer layers on GaN coatings on silica NS will now be addressed.

**Figure 6:**
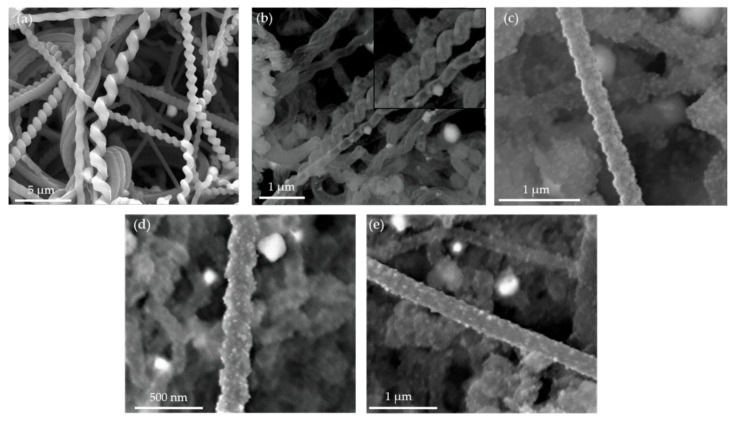
SEM Images of (**a**) as-grown silica nanospring (NS) ensemble showing smooth features, (**b**) 50 cycles GaN on NS with relatively smooth features and little indication of crystalline growth. (**c**) 100 cycles GaN on NS showing clear crystalline growth and NS coated with 50 cycles GaN with buffer layers of (**d**) AlN and (**e**) Al_2_O_3_/AlN showing crystallite growth. The large white particles are gold catalyst used during the NS growth process [47].

**Figure 7:**
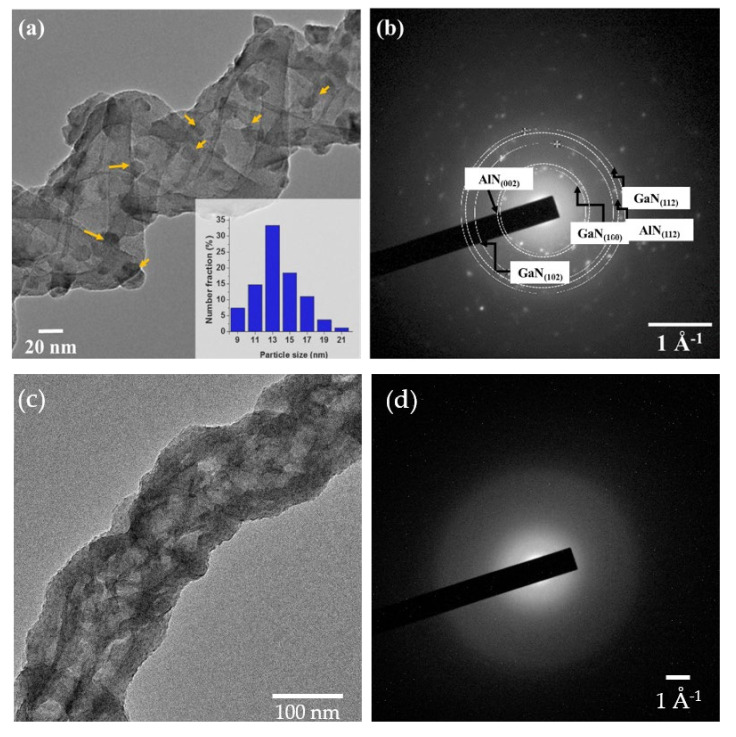
(**a**) Transmission Electron Microscopy (TEM) micrograph of a 50 cycle GaN coating with an Al_2_O_3_/AlN buffer layer on a silica NS. The yellow arrows indicate GaN nanoparticles. The inset is the GaN nanoparticle size distribution, (**b**) Selected Area Electron Diffraction (SAED) pattern obtained on the region consisting of rings corresponding to GaN and the AlN buffer layer, (**c**) a TEM micrograph of a 50 cycle GaN coating on a silica NS and (**d**) the corresponding SAED pattern, where diffraction spots are not observed.

SEM has been used to investigate of the morphology of GaN on NS, as opposed to AFM for obvious reasons. Figure 6a is an ensemble of NS structures as grown with no coatings, note the smooth surfaces that is characteristic of the amorphous structure. Figure 6b is an SEM image of 50 ALD cycles of GaN, which is characterized by small crystallites spread sparsely about the surfaces. By either increasing the number of ALD cycles (Figure 6c) or the addition of buffer layers (Figure 6d,e), there is a clear shift from smaller to larger crystallites. TEM studies further complement this analysis, where Figure 7 shows TEM micrographs and SAED patterns. The TEM micrograph in Figure 7a shows the distribution of GaN crystallites (marked by yellow arrows) for 50 cycles of GaN on a NS with Al_2_O_3_/AlN buffer layers. The average size of the GaN nanocrystals is 11.5 ± 0.5 nm, where the size distribution is summarized in the inset of Figure 7a. Figure 7b is the corresponding SAED pattern. The SAED patterns were indexed using standard hexagonal GaN (h-GaN) and AlN (h-AlN) phases, having a space group of P6_3_mc. The lattice parameters of GaN (a = 3.195 Å and c = 5.182 Å) and AlN (a = 3.128 Å and c = 5.017 Å) were used for identifying the planar spacing [68,69]. The rings of the GaN SAED correspond to the (100), (102), and (112) reflections, respectively [70]. In addition, diffraction rings corresponding to (002) and (112) reflections of AlN are also present. Diffraction spots were not observed for SiO_2_ NS, as they are amorphous, nor Al_2_O_3_. Figure 7c is a TEM micrograph of 50 cycles of GaN on NS, where crystallites of GaN cannot be resolved. The absence of diffraction spots in the SAED of the GaN-NS in Figure 7d indicates that the GaN coating is amorphous. 

**Figure 8:**
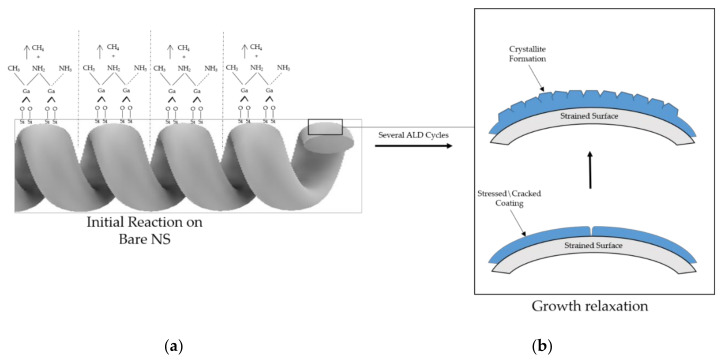
(**a**) Initial surface reaction trimethylgallium (TMG) with the bare NS, where Ga bonds to O sites on the surface of the NS and releases methane byproducts and (**b**) a schematic representation of GaN growth on the strained NS surface, where crystallite growth emerges concomitant with the release of internal strain.

In order to understand why one GaN coating is amorphous and the other polycrystalline, one needs to appreciate the effects of NS surface preparation (with or without buffer layers), as well as changes in the internal stress of the coating as a function of thickness. We propose that stress develops due to the helical morphology (curved surface) of the NS and the impact of different coefficients of thermal expansions, where the latter is significant with the high deposition temperature [17,34,71]. We begin by first examining the deposition of GaN on NS without a buffer layer. The initial ALD reaction is at SiO_2_ sites, where a layer of Ga_2_O_3_ forms prior to GaN formation. Initially, TMG decomposes to form Ga-O bonds, concomitant with the release of methane. This is schematically illustrated in Figure 8a [72,73,74,75]. Eventually, Ga_2_O_3_ deposition transitions to GaN. The initial layers of GaN are amorphous due to the lack of periodicity of the surface of the amorphous silica NS and the curvature of the NS surface. Consequently, the Ga bonds will be highly distorted, thereby producing internal strain. After these initially strained amorphous layers, the internal strain relaxes and GaN crystallites begin to form, which is illustrated in the model in Figure 8b, and subsequently leads to the polycrystalline coating in Figure 7a. The conclusion is that during growth, the GaN bonds are highly distorted, where internal stress is alleviated by the amorphous structure of thin GaN coatings (≤50 nm). Eventually, with sufficient release of internal strain in the coating, amorphous growth transitions to polycrystalline growth. In the present study, this appears to occur between 50 and 100 cycles (Figure 6c). 

With the introduction of AlN and Al_2_O_3_/AlN buffer layers prior to HTALD deposition of GaN, we see crystallite formation for ≤50 cycles. This is attributed to better lattice matching relative to the silica surface of the nanosprings and indexing of GaN to the buffer layers, which ultimately reduces internal strain within the coating. It has been demonstrated that AlN and Al_2_O_3_ interlayers relieve tensile stress during the initial stages of GaN TF growth [76,77]. We suspect that buffer layers are particularly critical for coating silica nanosprings because of the differences between the lattice parameters and the coefficients of thermal expansions of GaN and SiO_2_ [44,66]. Note that the reduction in internal strain of the GaN coating associated with buffer layers is only useful for the initial layers due to the continued thermal and tensile stresses present during growth. Furthermore, the curvature of the surface of the NS can lead to a crossover from internal strain to stress or vice a versa during the initial phase of GaN deposition. 

**Figure 9:**
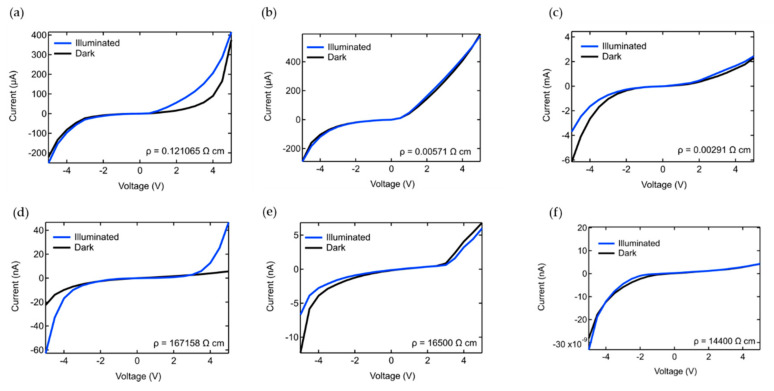
IV curves of (**a**–**c**) thin films of GaN, AlN/GaN, and Al_2_O_3_/AlN/GaN and (**d**–**f**) NS coated with GaN, AlN/GaN, and Al_2_O_3_/AlN/GaN, respectively. Measurements were taken with 470 nm light (blue) and dark (black) conditions. Resistivity measurements under dark conditions have for each sample have been included.

While the stoichiometry, crystallinity, and morphology of the GaN coatings on 1D nanostructures like silica NS are important, the electrical properties of the core-shell material ultimately determines their usefulness. Consequently, the IV characteristics of a mat of GaN coated NS and equivalent GaN TF have been tested and compared. This allows one to determine which electrical characteristics are associated with an assembly of 1D nanostructures and which are specific to the electrical properties of the GaN, which in turn depend on the morphology and stoichiometry of the coating. The IV curves of the GaN thin films with and without buffer layers under 470 nm illuminated and dark conditions are displayed in Figure 9a–c and those for corresponding GaN coated NS are displayed in Figure 9d–f. The GaN TF are n-type with relatively low resistivities based on their IV curves and the literature [78,79,80]. The IV curves for each TF sample are nonlinear and exhibit Schottky characteristics. The Schottky nature of the contacts arises from the use of indium beads, as opposed to traditional metal deposited contacts, which were chosen because of the incompatibility of metal deposited contacts with the NS mats and for the sake of consistency between the measurements of the two types of samples. The dark IV curve of GaN TF on Si in Figure 9a is symmetric about the origin, but under illumination is asymmetric with a forward bias turn-on voltage of 0.5 V, which is similar to reported values [81,82]. The dark IV curve for GaN/NS is also fairly symmetric but with a flatter profile. However, under illumination it displays symmetric turn-on voltages of 4 V in either forward or reverse bias. Note that the current is in the uA range for the GaN TF and nA for the GaN/NS. The significantly higher resistivity of NS samples relative to their TF counterparts is typical [35,83]. The general characteristics of the dark and illuminated IV curves of the GaN/NS samples exhibit similarities to their TF counterparts, as well as differences. As we will show, this is likely a consequence of the geometric differences between the two supports.

With the addition of the AlN and Al_2_O_3_/AlN buffer layers the characteristics of the NS and TF samples are nearly identical. Take for example GaN grown on an AlN buffer layer in Figure 9b,e for the TF and NS. With the exception of the onset voltage in forward bias and a slight photo-response of the NS sample, both exhibit steep onsets in forward bias and gradual onsets in reverse bias. This suggests that buffer layers produce similar forms of GaN, independent of the substrate, ergo, one can obtain GaN coatings on 1D nanostructures and thin films with equivalent electrical characteristics by using an AlN buffer layer. The IV curves of the TF and the NS with AlN/Al_2_O_3_ buffer layers are again quite similar, with the exception of a photo-response for the TF and a lack of one for the NS sample. Once again, the consistency between the TF and NS samples suggests that the buffer layer equivalently impacts the GaN, regardless of the substrate. The lack of or minimal photo-response with buffer layers is consistent with studies of GaN/AlN superlattices, where the photo-response either disappears or is significantly reduced [84,85]. Furthermore, the decreasing resistivity of the TF and the NS samples with the addition of buffer layers is indicative of improved GaN crystallinity due to a reduction of defects based on reports of decreasing sheet resistances of GaN thin films and heterostructures with the inclusion of buffer layers that has been attributed to improved crystallinity [86]. Note that the photo-response of GaN is dependent upon the quality of crystallinity, where the photoionization of electrons from deep levels drives the materials photoconductivity. Recombination centers act as deep acceptors, ergo, with less defects contributing to compensation from interlayers, the photo-response diminishes [87]. Gallium vacancies (V_Ga_) are believed to be the principle point defect in this process, hence, minimizing their concentration decreases the photoconductivity [88]. This mechanism also explains why the resistivity of the HTALD GaN in this study is lower with the inclusion of buffer layers, where less compensation from point defects and dislocations gives way to higher conductivity. We further suggest that the inclusion of buffer layers reduces threading dislocations (TD) that have been shown to increase the resistivity of GaN [89]. Edge-type TD escalate compensation in GaN, which increases its resistivity and photoconductivity [90]. The conclusion is that the decrease in the resistivities and photoconductivities of HTALD GaN coatings with buffer layers, regardless of the substrate, is indicative of improved crystallinity and a reduction in the concentrations of defects.

The n-type conductivity of the GaN coatings and the observation of oxygen in the XPS spectra (Figure 3) indicates that oxygen is acting as an n-type dopant, where it is generally accepted that oxygen substitutes into nitrogen vacancies (V_N_), acting as an uncompensated shallow donor and the main contributor to n-type GaN conductivity [91,92,93,94,95,96]. These oxygen atoms are compensated, where ~10% are electrically active, which produces unintentionally doped n-type GaN when V_Ga_ are kept relatively lower than the concentration of oxygen donors [97]. The concentration of oxygen in the sample in this study are in the range of 10^16^/cm^3^–10^21^/cm^3^. With high concentrations one should observe surface abnormalities, such as cracking and pitting [98],which are not present in the AFM images in Figure 5.

## 5. Conclusions

We have successfully designed and tested a novel high-temperature ALD reactor for the growth of GaN thin films and 3D coatings on silica nanosprings. XPS analysis indicates that there are low concentrations of C and O in the GaN films. The (002) and (102) Bragg peaks of GaN are observed in the HRXRD spectra, where the (102) peak is attributed to stress in the films and indicate that the thin film growth is in the c-axis direction. The effects of buffer layers of AlN and Al_2_O_3_/AlN on the morphology of the GaN thin films and GaN coatings on silica nanosprings have also been examined. AFM showed that the buffer layers decreased the RMS roughness of the GaN thin films. SEM and TEM demonstrated that the inclusion of buffer layers improved the crystallinity of GaN deposited onto silica nanosprings, where the improvement was qualitatively explained in terms of a stress/strain relaxation model that accounted for the curvature of the surface of the nanosprings, thermal stress, and stress during the initial phase of deposition. Finally, the comparisons of the electrical properties of the GaN thin films with GaN coated nanosprings with and without buffer layers were found to exhibit similar characteristics. The inclusion of buffer layers decreased the resistivity and photoconductivity of the GaN thin films and the GaN coated nanosprings, which for both types of samples is attributed to improved crystallinity and lower concentrations of defects.
